# "Splicing" the Urethra

**Published:** 1898-04-30

**Authors:** 


### "SPLICING" THE URETHRA.

By giving a new reason for an old operation, and by means of a somewhat taking name, Mr. Reginald Harrison brings up again our old friend, the internal urethrotomy of Maissoneuve. We used to look upon this, and, in fact, Maissoneuve himself looked upon it, as a means of dividing a stricture. Mr. Harrison, however, regards it as a means of putting a splice into the urethra. In cases which will not yield to dilatation alone we may, he says, do much good, and prevent the bad effects of back pressure, gradually extending to the bladder and kidneys above, "by introducing a splice of new material into the contracted portion of the canal, much on the same principle as we would expand a tight garment." As healing takes place, under the occasional use of a bougie, a splice of new tissue is introduced between the lips of the incision, and thus the calibre of the canal may be considerably increased in a short time; all of which is not only old but interesting. What is new is that an operation of this sort may be made more successful and less dangerous by antiseptic methods—by washing out the bladder with a solution of perchloride of mercury (1 in 6,000) until the lotion returns quite clear; by giving boric acid, or, better still, "boricite," by the mouth for a couple of days or so before the operation is done; and by great care in asepticising the instruments. As Mr. Harrison says, "the extension of the antiseptic principle to these operations has in all respects greatly benefited them." If it should turn out that, under the antiseptic methods which Mr. Harrison advocates, the "splice" introduced is non-contractile, there would be no possible doubt as to the enormous advantage of the proceeding.

